# Life expectancy among women and men with HIV after achieving sustained virologic suppression in the Bronx, New York: a clinical cohort study

**DOI:** 10.1016/j.lana.2026.101668

**Published:** 2026-10-14

**Authors:** Brandilyn A. Peters, Melissa Fazzari, Uriel R. Felsen, Heidi E. Jones, Mindy S. Ginsberg, David B. Hanna, Kathryn Anastos

**Affiliations:** a Department of Epidemiology and Population Health, Albert Einstein College of Medicine, Bronx, NY, USA; b Department of Medicine, Albert Einstein College of Medicine, Bronx, NY, USA; c City University of New York Graduate School of Public Health and Health Policy (CUNY SPH), New York, NY, USA

**Keywords:** HIV, Life expectancy, Bronx, Sex difference, Virologic suppression, Mortality

## Abstract

**Background:**

Whether female lifespan advantage is maintained among people with virologically suppressed HIV is unclear. Localized cohorts with demographically similar women and men may reveal associations obscured nationally. We estimated sex differences in life expectancy among people with HIV (PWH) in the Bronx, NY, after first achieving sustained virologic suppression.

**Methods:**

Among PWH receiving care in Montefiore Health System in the Bronx, NY who achieved sustained virologic suppression for ≥1 year (1997–2021), follow-up began at the end of the earliest qualifying window (index date) and ended at death or 2023. A Poisson model estimated mortality rate ratios by age, sex, race/ethnicity, HIV acquisition category, CD4+ T-cell count, and health insurance at the index date; age-specific mortality rates estimated remaining life-years for a 30-year-old using Chiang’s life table method.

**Findings:**

PWH (n = 9999; 4075 [41%] women, 8392 [84%] Black or Hispanic) were followed for a median of 10.7 years; 2675 (27%) died. Men had 32% higher mortality risk than women (rate ratio: 1.32; 95% CI: 1.21–1.43). Compared to Black (non-Hispanic) PWH, Hispanic PWH had 19% lower mortality risk (RR: 0.81; 95% CI: 0.74–0.88), while those with White or other/unknown race/ethnicity had higher mortality risk. Among PWH with public insurance and CD4+ T-cell count ≥500 cells/μL, remaining life-years at age 30 were 45 and 41 for Hispanic women and men with heterosexual acquisition, 42 and 38 for Black women and men with heterosexual acquisition, and 44 and 41 for Hispanic and Black men with MSM acquisition.

**Interpretation:**

Women with HIV in the Bronx after sustained virologic suppression lived approximately 4 years longer than comparable men, approaching sex differences in the general population. Local, disaggregated population analyses may help clarify sex differences in longevity among PWH, thereby informing clinical and public health priorities.

**Funding:**

NIH.

**Research in context**

**Evidence before this study**

We examined literature in PubMed (database inception – June 2026) that examined life expectancy by sex among people with HIV in North America or Europe, which did not restrict follow-up based on time after antiretroviral therapy (ART) initiation, using search terms “HIV”, “mortality”, “survival”, and “life expectancy”. Life expectancy did not differ by sex in a North American AIDS Cohort Collaboration on Research and Design study of people with HIV starting ART, a Kaiser Permanente analysis of insured U.S. adults with HIV, or a meta-analysis of people with HIV starting ART in high-income countries. In U.S. studies using surveillance data of people diagnosed with HIV, one study reported longer life expectancy for men than women, while others (one national and one in New York City) showed that sex difference in life expectancy varies by race/ethnicity. In people with HIV starting or already on ART in the Antiretroviral Therapy Cohort Collaboration and/or UK Collaborative HIV Cohort Study, women had higher life expectancy than men by 1–3 years depending on the sub-group. Observational studies in Canada of people with HIV starting ART have shown longer life expectancy for men than women. Most of these study populations were composed of 70–80% men. Taken together, these studies showed a diminished or non-existent longevity advantage in women compared to men with HIV, unlike the ∼5 years of longer life that women experience on average in the general population.

**Added value of this study**

Differences in social determinants of health between women and men with HIV, that exist both in the U.S. and internationally, may obscure effects of sex on life expectancy in people with HIV. Highly localized populations of people with HIV with demographically similar women and men, such as in the Bronx, NY, may provide a better framework to examine sex differences in life expectancy for people with HIV on ART. We estimated life expectancy for people with HIV in the Bronx after they first achieved sustained virologic suppression, disaggregating estimates by sex, race/ethnicity, HIV acquisition category, CD4+ T-cell count, and health insurance type. Women had 4 years greater life expectancy than comparable men, while men with MSM acquisition had estimated life expectancy similar to women of the same race/ethnicity in the largest representative subgroups. To our knowledge, this is the longest female life expectancy advantage reported among people with HIV in a published study from North America or Europe.

**Implications of all the available evidence**

After first achieving sustained virologic suppression, women with HIV in the Bronx maintain a similar life expectancy advantage as women in the general population, suggesting that HIV infection in the setting of virologic suppression may not override the advantage of female sex in longevity in this setting. Prior reports of non-existent or diminished life expectancy advantage for women with HIV may partly reflect differences in social determinants of health for women and men with HIV on wider geographic scales, along with differences in study design, cohort composition, and analytic approach. These findings highlight the value of life expectancy comparisons within highly local populations of people with HIV.

## Introduction

Life expectancy for people living with HIV (PWH) has increased dramatically due to improvements in antiretroviral therapy (ART).^1^ Since the advent of highly active combination ART in 1996, successive improvements in ART regimens have resulted in better suppression of HIV replication, less toxicity, fewer side-effects, decreased drug resistance, and reduced pill burden.^2^ In the modern era, life expectancy for PWH on ART in North America and Europe is approaching that of the general population, though still lagging behind by several years.^3^^,^^4^

In almost all human populations, including the general U.S. population, women on average live longer than men (by approximately 5 years globally and in the U.S.).^5^ However, it is not clear whether this life expectancy advantage is retained for women vs. men with HIV on ART. Large collaborative cohort pooling projects from Europe and North America have reported a diminished lifespan advantage of only 1–3 years for women compared to men with HIV, depending on the sub-group (e.g., by year of ART initiation, HIV acquisition category, CD4+ T-cell count, and virologic suppression).^3^^,^^6^^,^^7^ Strikingly, other studies in the U.S. and Canada have shown that life expectancy may not differ or may even be higher for men living with HIV (MWH) than for women living with HIV (WWH) across different population types (e.g., those starting ART, or those with HIV diagnosis).^8^, ^9^, ^10^, ^11^, ^12^, ^13^ A meta-analysis of 8 cohort studies indicated that for PWH initiating combination ART in high-income countries, life expectancy did not differ between women and men.^14^ Life expectancy comparisons for women and men with HIV on a wide geographic scale (i.e., national or international level) may be hindered by sociodemographic differences in the HIV epidemic between women and men by acquisition route, race/ethnicity, and socioeconomic status,^15^ posing a challenge to disentangle social determinants of health from effects of sex. For example, differences in access to health care and ART adherence could drive differences in life expectancy by sex. Therefore, comparisons of life expectancy by sex may be most interpretable when made within similar covariate-defined groups, including groups defined by sociodemographic characteristics and HIV acquisition routes.

Here, we sought to examine life expectancy for PWH on a hyper-local level, among individuals who had achieved sustained virologic suppression while receiving care through the Montefiore Health System in the Bronx, New York (NY). The diverse population of the Bronx, comprised of >85% minoritized (Hispanic or Black) people, experiences the highest rate of new HIV infections among all New York counties, and the 6th highest among all counties nationally.^16^ In the Bronx, 96% of new HIV diagnoses occur in Blacks or Hispanics, and 27% occur in women,^17^ a high proportion of women compared to 19% of new cases occurring in women nationally. In this hyper-local setting with high sociodemographic comparability between women and men, we aimed to analyze life expectancy after patients first achieved sustained virologic suppression, using demographic and clinical characteristics measured at the index date, to examine if women maintain their usual longevity advantage among a population well-treated with ART.

## Methods

### Study population

The Montefiore Health System (MHS) is an integrated health care system with 4 hospitals and >21 ambulatory care sites in the Bronx. The population that MHS serves is predominantly Black and Hispanic, reflecting the two largest racial/ethnic demographic groups in the Bronx. Data were extracted from the Montefiore Einstein HIV Clinical Cohort,^18^ which contains data on PWH and people without HIV who receive care within MHS and combines data from the electronic health record, including inpatient and outpatient visits; laboratory test results; prescription data; and intake data from New York State’s AIDS Institute Reporting System (AIRS).

PWH aged 18 or older receiving care at MHS in the Bronx from 1/1/1997 to 12/31/2021, with at least two viral load measurements obtained at least 30 days apart and who achieved sustained virologic suppression as defined below, were included (Supplementary Fig. S1). A threshold of <1000 copies/mL was used to define virologic suppression to allow a consistent definition across the full 1997–2021 study period, during which viral load assay sensitivity and reporting thresholds changed over time. The date of earliest sustained virologic suppression, hereafter referred to as the index date, was defined retrospectively as the end of the earliest one-year window where at least 80% of all viral load measurements were suppressed (<1000 copies/mL). Among 15,277 patients with confirmed HIV, 10,608 patients were found to meet the criteria and thus had a valid index date. Deaths were primarily ascertained through linkage with the National Death Index (NDI)^19^ through 12/31/2023. The NDI captures deaths occurring in the U.S. and dependent territories using death certificate data submitted by all state vital records offices. Deaths documented in the electronic health record (EHR), with or without linkage to the NDI, were also included. Patients not identified as deceased through the NDI or EHR were administratively censored on 12/31/2023. Follow-up began at the index date and ended at death or administrative censoring on 12/31/2023. After cohort entry, participants remained under follow-up regardless of subsequent ART interruption, loss of virologic suppression, or absence of later visits within MHS (i.e., no censoring). We excluded from the analysis data set patients missing CD4+ T-cell count at the index date (n = 249), patients with HIV acquisition category classified as perinatal acquisition (mother with or at risk of HIV, n = 151), and patients who were reported to have died but whose dates of death were unknown (n = 27) or died during the one-year window (n = 182). Following these exclusions, a total of 9999 PWH were included in this analysis, among whom 2675 died during follow-up. The majority of the deaths (2,672, 99.9%) were captured by the NDI, while 3 deaths ascertained from the EHR were not linked to the NDI.

### Patient data

Patients were categorized by calendar period of the index date as follows: 1997–2001, 2002–2006, 2007–2011, 2012–2016, or 2017–2021. We considered data on sex at birth, race/ethnicity (Black, White, Hispanic, or other/unknown), age at index date (18–19, 20–29, 30–39, 40–49, 50–59, 60–69, 70–79, 80+ years), CD4+ T-cell count at index date (<200, 200–349, 350–499, ≥500 cells/μL), HIV acquisition category (heterosexual, men who have sex with men [MSM], injection drug use [IDU]-related HIV acquisition, other [includes potential blood product or unconfirmed sexual activity acquisition], or unknown), and health insurance type at index date (commercial, public, or other/unknown). Commercial insurance included private employer-sponsored or individually purchased health plans; public insurance included Medicare (federal program primarily for adults aged ≥65 years and some younger people [including those living with HIV] with long-term disabilities or end-stage renal disease) and Medicaid (joint federal-state program for eligible people with low income); and other/unknown included self-pay, unclassified, unknown, or missing insurance status. Patients without documented commercial or public insurance were included in the other/unknown category and were not excluded from the analysis on the basis of insurance status. Underlying causes of death were categorized using ICD-10 codes.

### Ethics

This study follows the Declaration of Helsinki and was granted a waiver of informed consent by the Albert Einstein College of Medicine Institutional Review Board (IRB number: 2024–15638).

### Statistical analysis

The study was conducted from January 2024 to June 2026, including study design, data extraction, NDI linkage, analysis, and manuscript preparation. All computations were done using SAS version 9.4. A Poisson regression model including an offset for years of follow-up was used to estimate mortality rate ratios and 95% confidence intervals. The variables included in our *a priori* model were calendar period of the index date, age, sex at birth, race/ethnicity, CD4+ T-cell count, HIV acquisition category, and health insurance type. For model parsimony and to avoid unnecessary stratification, the final multivariable Poisson model included only covariates that were statistically significant at the 0.05 level based on Type III Wald tests.

Using the parameter estimates from the final multivariable Poisson model, expected remaining years of life were thus estimated for a representative 30-year-old patient. Remaining life-years were computed using age-specific mortality rates estimated from the final Poisson regression model. Mortality rates were estimated for pre-specified combinations of sex, race/ethnicity, health insurance type, HIV acquisition category, and CD4+ T-cell count groups. Age was categorized into eight 10-year intervals. For each bootstrap replicate, a Poisson regression model with a log link and offset for person-years at risk was fit to estimate age-specific mortality rates while adjusting for demographic and clinical covariates. Predicted mortality rates ($m_{x}$) for each age interval were generated from the fitted model for prespecified covariate patterns corresponding to the sub-group of interest. These age-specific rates were then converted into interval-specific probabilities of death ($q_{x}$) using: $q_{x}=1-e^{-m_{x}n}$, where n represents the width of the age interval (10 years in this analysis). Chiang’s life table method^20^ was then applied to these estimated death probabilities. A synthetic cohort of 1000 individuals was initialized at the start of the first age interval and carried forward across ten-year age bands. Within each interval, the number of deaths and survivors were estimated recursively based on the predicted probability of death. Person-years lived within each interval were calculated assuming deaths occurred uniformly throughout the interval, such that individuals who died contributed, on average, one-half of the interval length. Remaining life-years at the beginning of each age interval were computed by summing all person-years lived in that interval and all subsequent intervals and dividing by the number of individuals alive at the start of the interval. In the final age interval, all remaining individuals were assumed to die, with deaths assumed to occur uniformly throughout the 10-year interval. Uncertainty was quantified using nonparametric bootstrap resampling with 1000 replicates. For each replicate, the full modeling and life table estimation procedure was repeated, generating bootstrap distributions of remaining life-years estimates for each subgroup and age interval. Bootstrap percentile-based 95% confidence intervals were calculated from these empirical distributions.

Model assumptions for the Poisson regression analyses were evaluated by examining the adequacy of the log-linear functional form and evaluating the model for potential overdispersion. Pearson goodness-of-fit statistics indicated some overdispersion, however residual diagnostics indicated that this was primarily driven by a small subset of individuals with very low person-time contributions (<6 months). Sensitivity analyses were performed using negative binomial regression models, which allows for an additional dispersion parameter, and estimates were compared with the primary Poisson-based model to assess robustness. As results were substantively similar, the original Poisson modeling framework was retained. Sensitivity analyses were also performed excluding 89 COVID-related deaths (ICD-10 code U07.1) or 297 deaths from non-natural causes (ICD-10 codes U00–Y99 excluding U07.1).

### Role of the funding source

The funding source had no role in data collection, analysis, interpretation, or publication of study findings.

## Results

Among 9999 patients included in the analysis, 4075 (41%) were female and 8392 (84%) were Black or Hispanic (Table 1). At the index date, 7084 (71%) were on public insurance and 3765 (38%) had CD4+ T-cell counts ≥500 cells/μL. Among women, 3497 (86%) were Black or Hispanic, 2876 (71%) were on public insurance, 1655 (41%) had CD4+ T-cell counts ≥500 cells/μL, and 2788 (68%) and 462 (11%) reported acquisition risk from heterosexual activity or IDU, respectively. Among men, 4895 (83%) were Black or Hispanic, 4208 (71%) were on public insurance, 2110 (36%) had CD4+ T-cell counts ≥500 cells/μL, and 3820 (64%) and 1026 (17%) reported acquisition risk from sexual activity (1679 [28%] heterosexual and 2141 [36%] MSM) or IDU, respectively. Median follow-up time after the index date was 10.7 years (11.9 years in women and 9.8 years in men) (Table 2). Among 2675 deaths, cause-of-death distribution differed by sex and other factors (p ≤ 0.01; Supplementary Table S1).

**Table 1:** Characteristics at the time of first achieving sustained virologic suppression for people with HIV receiving care in the Montefiore Health System in the Bronx, NY, 1997–2021 (n = 9999).

	All (n = 9999)	Female (n = 4075)	Male (n = 5924)
	N (%)	N (%)	N (%)
Year of earliest sustained viral suppression			
1997–2001	2000 (20.0)	993 (24.4)	1007 (17.0)
2002–2006	1852 (18.5)	781 (19.2)	1071 (18.1)
2007–2011	2229 (22.3)	946 (23.2)	1283 (21.7)
2012–2016	2282 (22.8)	855 (21.0)	1427 (24.1)
2017–2021	1636 (16.4)	500 (12.3)	1136 (19.2)
Age, years			
18–19	147 (1.5)	59 (1.4)	88 (1.5)
20–29	1124 (11.2)	364 (8.9)	760 (12.8)
30–39	2266 (22.7)	978 (24)	1288 (21.7)
40–49	3174 (31.7)	1363 (33.4)	1811 (30.6)
50–59	2378 (23.8)	966 (23.7)	1412 (23.8)
60–69	755 (7.6)	281 (6.9)	474 (8.0)
70–79	138 (1.4)	59 (1.4)	79 (1.3)
80+	17 (0.2)	5 (0.1)	12 (0.2)
Race/ethnicity			
Black, non-Hispanic	4353 (43.5)	1913 (46.9)	2440 (41.2)
Hispanic, any race	4039 (40.4)	1584 (38.9)	2455 (41.4)
Other/Unknown	1099 (11.0)	399 (9.8)	700 (11.8)
White, non-Hispanic	508 (5.1)	179 (4.4)	329 (5.6)
CD4+ T-cell count, cells/μL			
<200	2129 (21.3)	757 (18.6)	1372 (23.2)
200–349	2154 (21.5)	878 (21.5)	1276 (21.5)
350–499	1951 (19.5)	785 (19.3)	1166 (19.7)
≥500	3765 (37.7)	1655 (40.6)	2110 (35.6)
HIV acquisition category			
Heterosexual	4467 (44.7)	2788 (68.4)	1679 (28.3)
Injection drug use-related^a^	1488 (14.9)	462 (11.3)	1026 (17.3)
Men who have sex with men	2141 (21.4)	N/A	2141 (36.1)
Other/Unknown^b^	1903 (19.0)	825 (20.2)	1078 (18.2)
Health insurance type			
Commercial	1006 (10.1)	343 (8.4)	663 (11.2)
Public	7084 (70.8)	2876 (70.6)	4208 (71.0)
Other/unknown	1909 (19.1)	856 (21.0)	1053 (17.8)

a Includes both injection drug use-related acquisition and men who have sex with men (n = 74).

b Other HIV transmission category includes potential blood product exposure and unconfirmed sexual activity transmission.

**Table 2:** Follow-up and deaths after first achieving sustained virologic suppression for people with HIV receiving care in the Montefiore Health System in the Bronx, NY, 1997–2021 (n = 9999).

	All (n = 9999)	Female (n = 4075)	Male (n = 5924)
Follow-up, y, median (IQR)	10.7 (5.6, 16.7)	11.9 (6.5, 18.5)	9.8 (5.1, 15.3)
Deaths during follow-up, N	2675	989	1686
Age at death, y, median (IQR)	57.1 (50.6, 63.9)	56.4 (50.0, 63.6)	57.5 (51.0, 64.1)

The calendar period of the index date was not statistically significant in the initial multivariable Poisson model (Type III p = 0.15; Supplementary Table S2) and was thus not retained in the final model presented. Based on the final multivariable Poisson regression model for mortality, men had 32% higher mortality risk than women (rate ratio [RR] = 1.32, 95% CI: 1.21,1.43) (Table 3). Compared to Black non-Hispanic PWH, Hispanic PWH had 19% lower mortality risk (RR = 0.81, 95% CI: 0.74,0.88), while White non-Hispanic PWH or those with other or unknown race/ethnicity had higher mortality risk (White: RR = 1.20 [1.03,1.40], other/unknown: RR = 1.27 [1.13,1.44]). Older age, lower CD4+ T-cell count, and non-commercial health insurance (public or other/unknown, vs. commercial insurance) were associated with higher mortality risk (Table 3). Compared to the heterosexual acquisition category, IDU-related HIV acquisition or other factors were associated with higher mortality risk, while MSM acquisition was associated with lower mortality risk (Table 3). We observed a statistically significant interaction of sex with HIV acquisition category (p = 0.003), and in sex-stratified models, IDU-related HIV acquisition vs. heterosexual acquisition was more strongly associated with mortality among women (RR = 2.30; 95% CI: 1.97, 2.70) than among men (RR = 1.66; 95% CI: 1.47, 1.88) (Supplementary Table S3); we did not observe significant interactions of sex with age (p = 0.18) or other factors (Supplementary Table S3). In age-specific contrasts from the interaction model, male mortality was consistently significantly higher than female mortality among adults aged 30–79 years, but not among those aged <30 years or ≥80 years, where deaths were fewer and estimates were more imprecise (Supplementary Table S4). Sensitivity analyses excluding deaths related to COVID-19 or non-natural causes did not materially alter model estimates.

**Table 3:** Estimated mortality rate ratios in people with HIV in the Bronx, NY after first achieving sustained virologic suppression.

	Relative Risk (95% CI)	p-value
Sex at birth		
Male	1.32 (1.21,1.43)	<0.0001
Female	Reference	
Age, years^a^		
18–19	0.25 (0.13,0.48)	<0.0001
20–29	0.31 (0.25,0.4)	<0.0001
30–39	0.65 (0.57,0.73)	<0.0001
40–49	Reference	
50–59	1.71 (1.55,1.87)	<0.0001
60–69	2.5 (2.23,2.88)	<0.0001
70–79	3.50 (2.7,4.53)	<0.0001
80+	10.63 (6.46,17.49)	<0.0001
Race/ethnicity		
Hispanic, any race	0.81 (0.74,0.88)	<0.0001
Other/unknown	1.27 (1.13,1.44)	<0.0001
White, non-Hispanic	1.20 (1.03,1.40)	0.02
Black, non-Hispanic	Reference	
CD4+ T-cell count, cells/μL^a^		
<200	1.54 (1.39,1.7)	<0.0001
200–349	1.14 (1.02,1.26)	0.02
350–499	1.09 (0.97,1.22)	0.15
≥500	Reference	
HIV acquisition category		
Injection drug use-related	1.88 (1.7,2.07)	<0.0001
Men who have sex with men	0.78 (0.68,0.9)	0.001
Other/unknown	1.33 (1.2,1.48)	<0.0001
Heterosexual	Reference	
Health insurance type^a^		
Public	1.96 (1.66,2.31)	<0.0001
Other/unknown	1.40 (1.17,1.67)	0.0003
Commercial	Reference	

a At the index date.

To improve comparability, life expectancy estimates were disaggregated by sex, HIV acquisition category, race/ethnicity, health insurance type, and CD4+ T-cell count, allowing comparisons within otherwise similar covariate-defined groups. Remaining years of life were estimated for 30-year-old PWH after first achieving sustained virologic suppression, resulting in 336 life expectancy estimates for groups of PWH categorized by sex, race/ethnicity, HIV acquisition category, health insurance type, and CD4+ T-cell count at the index date (Supplementary Table S5; Fig. 1). Among the 336 covariate-defined groups included in Supplementary Table S5, the median group size was 11 participants (IQR: 4–29), 156 groups (46.4%) had fewer than 10 participants, and 104 groups (31.0%) had 0 or 1 observed death before further stratification by age. Therefore, interpretation focuses on the largest representative subgroups.

**Fig. 1:**
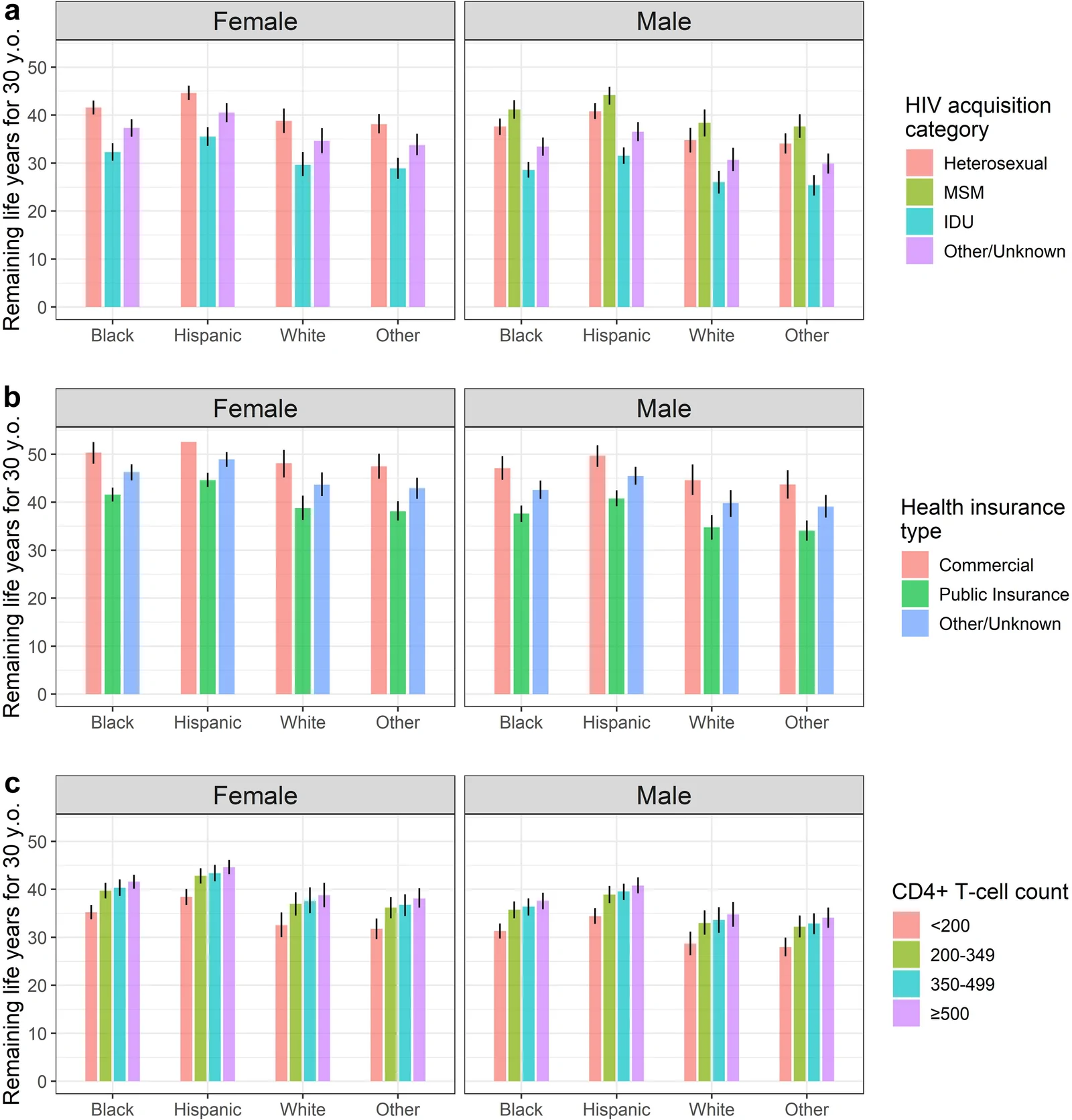
**Estimates of remaining years of life for 30-year-old people with HIV after first achieving sustained virologic suppression in the Bronx, NY.** Estimated remaining life-years with 95% confidence intervals are shown by sex and race/ethnicity, varying one covariate at a time: (a) HIV acquisition category, among people with public health insurance and CD4+ T-cell count ≥500 cells/μL at the index date; (b) health insurance type at the index date, among people with heterosexual HIV acquisition and CD4+ T-cell count ≥500 cells/μL; and (c) CD4+ T-cell count at the index date, among people with heterosexual HIV acquisition and public health insurance. The index date was defined as the date of earliest sustained virologic suppression. IDU, injection drug use-related HIV acquisition; MSM, men who have sex with men.

Among men, those with MSM acquisition risk had the highest life expectancies (Fig. 1a). Among women and men, those with commercial health insurance had the highest life expectancies, and those with CD4+ T-cell count <200 cells/μL had the lowest (Fig. 1b and c). Among all groups, the highest life expectancies were among Hispanic women and men with CD4+ T-cell count ≥500 cells/μL, commercial health insurance, and heterosexual or MSM acquisition risk, respectively (remaining life years [95% CI]: 52.6 [50.7,54.4] and 52.3 [50.0,54.5]) (Supplementary Table S5). Lowest life expectancy was observed for men of other/unknown race/ethnicity on public insurance with CD4+ T-cell count <200 cells/μL and IDU acquisition category (remaining life years [95% CI]: 20.3 [18.4,22.2]) (Supplementary Table S5). Across covariate-defined groups represented among both women and men, estimated remaining life-years were higher for women than comparable men by a median of 3.88 years (IQR: 3.74, 3.98).

We then considered the largest sub-groups for interpretation (Hispanic or non-Hispanic Black race/ethnicity, heterosexual or MSM acquisition, on public insurance and with CD4+ T-cell count ≥500 cells/μL at the index date), in order to highlight the most representative populations of PWH in the Bronx. Remaining life-years at 30 years of age were approximately 45 and 41 for Hispanic women and men with heterosexual acquisition category, respectively; 42 and 38 for Black women and men with heterosexual acquisition category, respectively; and 44 and 41 for Hispanic and Black men with MSM acquisition category, respectively (Fig. 1a). This equates to a total estimated lifespan of 75 and 71 for Hispanic women and men, and 72 and 68 for Black women and men, with heterosexual acquisition; and 74 and 71 for Hispanic and Black men with MSM acquisition (i.e., similar lifespan to female counterparts).

## Discussion

In this large study of women and men with HIV after first achieving sustained virologic suppression in the Bronx, NY, we found that WWH approached the life expectancy advantage observed in the general population, living 4 years longer than MWH with comparable characteristics (i.e., same race/ethnicity, HIV acquisition category, health insurance type, and CD4+ T-cell count). Similarly, Hispanic PWH had longer life expectancy than non-Hispanic Black PWH, as also observed in the general population. Still, life expectancies in this Bronx population of PWH were lower than that reported in vital statistics for the U.S. and New York City. As contextual benchmarks, recent U.S. vital statistics estimates^21^ indicate that in 2023, total estimated lifespan was 84 and 78.5 years for U.S. Hispanic women and men, approximately 8–9 years longer than Hispanic women and men with HIV in the Bronx; and 77.6 and 70.3 years for U.S. Black women and men, approximately 2–6 years longer than Black women and men with HIV in the Bronx. In 2021 in New York City,^22^ total estimated lifespan was 80.7 years (83.5 and 77.8 years for women and men; 81.2 and 76.1 for Hispanics and Blacks; 77.2 in Morrisania [Bronx neighborhood with the lowest life expectancy]), longer lifespans than our estimates for PWH in the Bronx. As a crude mortality comparison, the mortality rate in our cohort was 2336 deaths per 100,000 person-years (2675 deaths over 114,503 person-years), compared with 748 deaths per 100,000 population in the Bronx general population in 2023.^23^ These comparisons are descriptive only because the general-population estimates are based on period mortality rates, whereas follow-up in our cohort began as early as 1997, and because the cohort and general Bronx population differ in age structure, HIV status, and calendar period. However, the observed gap in life expectancy for Bronx PWH is in accordance with national and international estimates that life expectancy for PWH is lower than that of the general population.^3^

Previous studies of PWH in Europe and North America have reported life expectancy estimates by sex, with results not always consistent across studies regarding female advantage in longevity. In an analysis of life expectancy after 2015 among PWH on ART for at least 1 year in Europe and North America, involving the Antiretroviral Therapy Cohort Collaboration and the UK Collaborative HIV Cohort Study, women and men starting ART after 2015 had an estimated total lifespan of 79 years and 77 years, compared to estimated lifespans of 86 and 81 for women and men in the corresponding general population, respectively,^3^ indicating a diminished lifespan advantage of 2 years for WWH vs. MWH. Even in the setting of virologic suppression, women had only 3 years greater life expectancy than comparable men.^3^ Other reports from the Antiretroviral Therapy Cohort Collaboration^7^ and the UK Collaborative HIV Cohort^6^ of people with HIV starting ART indicated higher life expectancy in women than men by ≤2 years. In a North American AIDS Cohort Collaboration on Research and Design (NA-ACCORD) study of PWH starting ART in the U.S. and Canada^8^ and a Kaiser Permanente analysis of insured adults with HIV in California,^11^ life expectancy did not differ between women and men. In U.S. studies using national surveillance data of people diagnosed with HIV (i.e., not considering ART use), one study reported longer life expectancy for men than women^9^ and another reported higher life expectancy for Black women vs. men, but not for Hispanic or White women vs. men.^24^ In Canada, observational studies of people with HIV starting ART (the British Columbia Centre for Excellence Drug Treatment Program and the Canadian Observational Cohort Collaboration) or with diagnosed HIV (Comparative Outcomes And Service Utilization Trends study) have shown longer life expectancy for men than women,^10^^,^^12^^,^^13^ with slower gains over time in life expectancy for women than men.^13^ Finally, a surveillance-based study of people diagnosed with HIV in New York City found longer life expectancy in 2018 for Hispanic and Black women (71 and 70.5) compared to Hispanic and Black men (68.9 and 67.9), respectively,^25^ similar to our results but with diminished lifespan advantage for women (<3 years). Differences between our study and prior studies may relate to different inclusion criteria, including our restriction to people who first achieved sustained virologic suppression, as well as the sociodemographic similarity of women and men in this local Bronx cohort, which may facilitate more comparable sex-specific estimates within this setting. Differences in ethnicity, migrant status, and HIV acquisition category may also contribute to differences between our findings and European cohort studies; for example, the UK Collaborative HIV Cohort included a high proportion of Black African women with heterosexual acquisition and White men with MSM acquisition,^6^ making sex-specific comparisons susceptible to confounding by social and structural factors. Interestingly, unlike the studies from North America and Europe, studies from sub-Saharan Africa have noted significantly greater life expectancy by 8–10 years in women compared to men with HIV starting ART,^14^ which may be driven by complex forces including better access and retention in care for WWH,^26^^,^^27^ and trends in the general population of longer life expectancy in women.^28^ Taken together, our findings highlight the value of local, disaggregated comparisons among demographically similar female and male populations as a complement to larger national and international studies. Our results strongly indicate that, among PWH in the Bronx after first achieving sustained virologic suppression, women have greater life expectancy than men, and furthermore that the female lifespan advantage is largely not diminished for PWH who achieve virological suppression, but may be similar to that in the general population (4 years longer life).

The mechanisms underlying women’s longer life expectancy are not fully understood even in the general population, and likely reflect a complex interplay of biological, behavioral, and social factors.^29^ Maintenance of virologic suppression is one possible pathway that could contribute to sex differences in mortality among people with HIV; however, a prior analysis of this Bronx clinical cohort found that virologic suppression and sustained virologic suppression were similar by sex,^18^ suggesting that differential virologic suppression or ART interruption is unlikely to explain the female life expectancy advantage observed here. Other factors, including smoking, alcohol use, substance use, comorbidities, social support, and other behavioral or social determinants of health, may also contribute to sex differences in mortality. We did not have consistently measured data on smoking, alcohol use, and substance use behaviors, and diagnosis-code-based measures of history of substance and alcohol use disorders may incompletely capture these exposures. Thus, residual confounding by these factors remains possible.

Previous U.S. studies of PWH have also shown differences in life expectancy by race/ethnicity, with inconsistent results. For example, in an NA-ACCORD analysis, life expectancy was higher for White PWH than non-White PWH^8^; in Kaiser Permanente^11^ and a national surveillance study of people with diagnosed HIV,^9^ life expectancy was higher for Hispanic PWH than non-Hispanic PWH. In another national surveillance study of people with an HIV diagnosis, life expectancy was higher for White MWH compared to Black or Hispanic MWH, and Black WWH compared to White or Hispanic WWH.^24^ The aforementioned analysis of people with diagnosed HIV in New York City indicated higher life expectancy for White compared to Black or Hispanic MWH, and Hispanic compared to Black or White WWH.^25^ Here, among PWH after achieving sustained virologic suppression, we observed that White PWH in the Bronx had lower life expectancy than Black and Hispanic PWH, and Hispanic PWH had the highest life expectancy, regardless of sex. The lower life expectancy for White PWH in the Bronx is likely reflective of the sociodemographic characteristics of the Bronx, a high poverty county where race/ethnicity may not reflect social determinants of health such as education, income, access to health care, social stigma, and marginalization in the same way it does on a citywide or national level. Our observation of higher life expectancy in Hispanic PWH is consistent with New York City and U.S. national vital statistics indicating longer life expectancy in Hispanic people. Importantly, our Poisson model adjusted for race/ethnicity, sex, and critical risk factors (HIV acquisition category, CD4+ T-cell count, and health insurance type), and our life expectancy estimates were disaggregated by these factors as well. This allowed for life expectancy estimates in disaggregated population groups for improved comparability, which may explain differences from previous literature in which groups were disaggregated by sex or race/ethnicity individually,^8^^,^^9^^,^^11^ or disaggregated by sex and race/ethnicity but not other factors.^24^^,^^25^

Our observation of higher life expectancy for men with HIV with MSM acquisition, and lower life expectancy for injection drug use-related acquisition, compared to heterosexual acquisition, is consistent with prior reports in national and international HIV populations.^3^^,^^7^, ^8^, ^9^^,^^24^ Men with MSM acquisition had higher life expectancy than men with heterosexual acquisition and, in some strata, estimates closer to those of women than to other men. This pattern suggests that HIV acquisition category may capture important differences in social context, healthcare engagement, comorbidities, behavioral risk factors, and other determinants of mortality, and underscores the importance of comparing life expectancy within covariate-defined groups and of considering social context when interpreting sex differences. Calendar period of the index date from 1997 to 2021 was not associated with mortality risk in the multivariable model, indicating that with achievement of virologic suppression and statistical adjustment for critical risk factors, estimated life expectancy remained constant over time for PWH in the Bronx.

Our study is strengthened by the large sample size from a diverse local population, 41% of whom were women, facilitating comparisons of demographically similar women and men from different racial/ethnic backgrounds; and by restricting to a population achieving virologic suppression, to facilitate better understanding of life expectancy among those with well-controlled HIV. We additionally adjusted our Poisson model for several additional important risk factors (HIV acquisition category, CD4+ T-cell count, and health insurance type), allowing for disaggregated and hence more comparable life expectancy estimates across groups. We also note several limitations: (1) Our study population of PWH receiving care from a single healthcare system may not represent the entire population of PWH in the Bronx, including individuals with substantial barriers to entering or remaining in HIV care, nor be generalizable to other populations. However, lack of commercial or public insurance would not necessarily preclude HIV care within Montefiore Health System because New York State has longstanding HIV Uninsured Care Programs, including ADAP and ADAP Plus, and clinic social work staff assist patients in obtaining coverage for HIV care and medications. (2) We did not have reliable information on first-generation migrant or immigration status, limiting direct comparison with European cohorts in which migrant status may differ substantially by sex. We also did not have complete validated data on all prior AIDS-defining conditions, which may capture HIV disease severity beyond CD4+ T-cell count; however, we adjusted for CD4+ T-cell count at the index date and report on HIV-related cause of death. (3) Deaths may have been missed if patients died outside the U.S. or if linkage to the NDI failed because of incomplete or inaccurate identifiers, name changes, common names, or use of alternate identifiers. Such missed deaths would tend to underestimate mortality; however, we have no reason to expect this under-ascertainment to differ systematically by sex, and therefore it is unlikely to explain the observed female life expectancy advantage. (4) While we assume that time of earliest sustained virologic suppression (i.e., the index date) represents entry into HIV care, we cannot account for patients receiving prior care (and virologic suppression) outside of the Montefiore healthcare system. Relatedly, we did not restrict or censor according to maintenance of virologic suppression during follow-up, nor did we account for time-varying CD4+ T-cell counts, thus our analysis reflects the reality of patients in care who may have periods where they are not virologically suppressed after achieving virologic suppression. Further, because follow-up began only after first achieving sustained virologic suppression, our estimates do not capture early mortality after ART initiation or mortality before or during the qualifying suppression window. Thus, our estimates should be interpreted as remaining life-years among PWH who survived to and achieved sustained virologic suppression in a real-world care setting, based on characteristics at that index date, rather than life expectancy from ART initiation or HIV diagnosis, or life expectancy during periods of continuous virologic suppression. (5) Finally, some covariate-defined groups used to generate life expectancy estimates were small and had few or no observed deaths. Although the Poisson model used information from the full cohort and bootstrap confidence intervals were used to quantify uncertainty, estimates for sparsely populated groups may be less stable and more dependent on model assumptions. Therefore, estimates for small strata should be interpreted cautiously, and our main interpretation emphasizes the largest representative subgroups.

In summary, life expectancy differences observed in the general population, specifically longer lifespans for women and Hispanics, were observed among PWH after first achieving sustained virologic suppression in the Bronx, NY. These findings, which differ from some reports on national and international scales, highlight the importance of comparisons within local populations with demographically similar women and men across racial/ethnic groups. It is reassuring to note that women with HIV in the Bronx after achieving sustained virologic suppression maintain a life expectancy advantage approaching that of women in the general population, suggesting that HIV infection may not override the potential advantage of female sex in longevity in this setting. Prior reports of non-existent or diminished life expectancy advantage for women with HIV^3^^,^^6^, ^7^, ^8^, ^9^, ^10^, ^11^, ^12^^,^^14^ may partly reflect differences in social determinants of health for women and men with HIV on wider geographic scales. Better understanding of disparities in life expectancy among PWH is crucial for clinical and policy decision-making to improve HIV care. Highly local and disaggregated population analysis, such as we have presented here, facilitates better understanding of life expectancy disparities, and should be performed in other local populations with high prevalence of HIV.

## Contributors

KA conceptualized the study; MSG and MF curated the data; MF and BAP accessed and verified the underlying data; MF conducted the data analysis; KA, BAP, MF, URF, HEJ, and DBH were involved in data interpretation; BAP performed data visualization and wrote the original draft; KA, MF, URF, HEJ, and DBH contributed to writing through review and editing. All authors revised and approved the final manuscript.

## Data sharing statement

Deidentified data and the study protocol can be made available upon reasonable request, after approval by the Einstein-Rockefeller-CUNY-Mount Sinai Center for AIDS Research (ERCM-CFAR)’s Clinical and Translational Science Core and relevant institutional review board.

## Declaration of interests

The authors report no conflicts of interest.
