# Supplementary material for: Life expectancy among women and men with HIV after achieving sustained virologic suppression in the Bronx, New York: a clinical cohort study
Source: Lancet Reg Health Am. 2026 Oct 14;65:101668. doi: 10.1016/j.lana.2026.101668 (PMC13683866; doi:10.1016/j.lana.2026.101668)
Supplement: Supplementary file 1 [file mmc1.pdf]

Life expectancy among women and men with HIV after achieving sustained virologic suppression in the Bronx, New York: a clinical cohort study

Brandilyn A. Peters, PhD, Melissa Fazzari, PhD, Uriel R. Felsen, MD, Heidi E. Jones, PhD, Mindy S. Ginsberg, BA, David B. Hanna, PhD, Kathryn Anastos, MD

Supplementary Table 1. Cause of death among decedents by subgroup

Cells show n (% of deaths within row). P-values compare cause-of-death distribution across levels of each subgroup.

Characteristic	Category	Deaths (n)	COVID-19	Cardiovascular disease	Cancer	HIV-related	Other/unknown natural causes	Substance use-related natural causes	Non-natural causes	P value
**Overall**	**Total**	2,675	89 (3.3)	492 (18.4)	443 (16.6)	775 (29.0)	519 (19.4)	60 (2.2)	297 (11.1)	
**Sex**	**Male**	1,686	57 (3.4)	312 (18.5)	259 (15.4)	486 (28.8)	317 (18.8)	40 (2.4)	215 (12.8)	0.01
Sex	Female	989	32 (3.2)	180 (18.2)	184 (18.6)	289 (29.2)	202 (20.4)	20 (2.0)	82 (8.3)	
**Age at index date, years**	**18-19**	9	0 (0.0)	0 (0.0)	0 (0.0)	5 (55.6)	0 (0.0)	0 (0.0)	4 (44.4)	<0.0001
Age at index date, years	20-29	71	3 (4.2)	4 (5.6)	10 (14.1)	15 (21.1)	16 (22.5)	2 (2.8)	21 (29.6)	
Age at index date, years	30-39	385	14 (3.6)	62 (16.1)	42 (10.9)	126 (32.7)	67 (17.4)	7 (1.8)	67 (17.4)	
Age at index date, years	40-49	904	30 (3.3)	150 (16.6)	146 (16.2)	280 (31.0)	157 (17.4)	28 (3.1)	113 (12.5)	
Age at index date, years	50-59	900	26 (2.9)	194 (21.6)	165 (18.3)	249 (27.7)	178 (19.8)	21 (2.3)	67 (7.4)	
Age at index date, years	60-69	328	14 (4.3)	58 (17.7)	64 (19.5)	86 (26.2)	83 (25.3)	2 (0.6)	21 (6.4)	
Age at index date, years	70-79	62	2 (3.2)	19 (30.6)	15 (24.2)	9 (14.5)	13 (21.0)	0 (0.0)	4 (6.5)	
Age at index date, years	80+	16	0 (0.0)	5 (31.2)	1 (6.2)	5 (31.2)	5 (31.2)	0 (0.0)	0 (0.0)	
**Calendar period of index date**	**1997-2001**	806	20 (2.5)	150 (18.6)	129 (16.0)	298 (37.0)	144 (17.9)	15 (1.9)	50 (6.2)	<0.0001
Calendar period of index date	2002-2006	647	18 (2.8)	114 (17.6)	113 (17.5)	199 (30.8)	123 (19.0)	22 (3.4)	58 (9.0)	
Calendar period of index date	2007-2011	616	20 (3.2)	109 (17.7)	110 (17.9)	152 (24.7)	137 (22.2)	14 (2.3)	74 (12.0)	
Calendar period of index date	2012-2016	453	21 (4.6)	90 (19.9)	70 (15.5)	98 (21.6)	83 (18.3)	9 (2.0)	82 (18.1)	
Calendar period of index date	2017-2021	153	10 (6.5)	29 (19.0)	21 (13.7)	28 (18.3)	32 (20.9)	0 (0.0)	33 (21.6)	
**Race/ethnicity**	**Black**	1,143	48 (4.2)	238 (20.8)	208 (18.2)	313 (27.4)	211 (18.5)	15 (1.3)	110 (9.6)	0.003
Race/ethnicity	Hispanic	996	27 (2.7)	162 (16.3)	160 (16.1)	287 (28.8)	206 (20.7)	26 (2.6)	128 (12.9)	
Race/ethnicity	Other/unknown	341	11 (3.2)	53 (15.5)	47 (13.8)	114 (33.4)	66 (19.4)	14 (4.1)	36 (10.6)	
Race/ethnicity	White	195	3 (1.5)	39 (20.0)	28 (14.4)	61 (31.3)	36 (18.5)	5 (2.6)	23 (11.8)	
**HIV acquisition category**	**Heterosexual**	1,061	37 (3.5)	214 (20.2)	188 (17.7)	298 (28.1)	202 (19.0)	16 (1.5)	106 (10.0)	0.0002
HIV acquisition category	IDU-related HIV acquisition	768	19 (2.5)	109 (14.2)	124 (16.1)	244 (31.8)	149 (19.4)	32 (4.2)	91 (11.8)	
HIV acquisition category	MSM	284	9 (3.2)	56 (19.7)	42 (14.8)	84 (29.6)	46 (16.2)	3 (1.1)	44 (15.5)	
HIV acquisition category	Other/unknown	562	24 (4.3)	113 (20.1)	89 (15.8)	149 (26.5)	122 (21.7)	9 (1.6)	56 (10.0)	
**Health insurance type**	**Commercial**	159	5 (3.1)	41 (25.8)	27 (17.0)	46 (28.9)	23 (14.5)	0 (0.0)	17 (10.7)	0.01
Health insurance type	Public	1,972	64 (3.2)	337 (17.1)	318 (16.1)	568 (28.8)	404 (20.5)	44 (2.2)	237 (12.0)	
Health insurance type	Other/unknown	544	20 (3.7)	114 (21.0)	98 (18.0)	161 (29.6)	92 (16.9)	16 (2.9)	43 (7.9)	
**CD4+ T-cell count**	**<200 cells/µL**	764	24 (3.1)	131 (17.1)	113 (14.8)	274 (35.9)	143 (18.7)	17 (2.2)	62 (8.1)	<0.0001
CD4+ T-cell count	200-349 cells/µL	621	19 (3.1)	112 (18.0)	101 (16.3)	193 (31.1)	113 (18.2)	14 (2.3)	69 (11.1)	
CD4+ T-cell count	350-499 cells/µL	490	15 (3.1)	85 (17.3)	76 (15.5)	141 (28.8)	97 (19.8)	11 (2.2)	65 (13.3)	
CD4+ T-cell count	≥500 cells/µL	800	31 (3.9)	164 (20.5)	153 (19.1)	167 (20.9)	166 (20.8)	18 (2.2)	101 (12.6)	

Supplementary Table 2. Estimated mortality rate ratios in people with HIV in the Bronx, NY after first achieving sustained virologic suppression (model includes calendar period).

	Relative Risk (95% CI)	P-value
**Calendar period[1]**		
1997-2001	1.12 (0.93,1.34)	0.24
2002-2006	1.00 (0.83,1.19)	0.97
2007-2011	0.99 (0.82,1.18)	0.87
2012-2016	0.99 (0.82,1.19)	0.90
2017-2021	Reference	
**Sex at birth**		
Male	1.32 (1.21,1.44)	<0.0001
Female	Reference	
**Age, years[1]**		
18-19	0.25 (0.13,0.47)	<0.0001
20-29	0.32 (0.25,0.4)	<0.0001
30-39	0.64 (0.57,0.72)	<0.0001
40-49	Reference	
50-59	1.74 (1.58,1.91)	<0.0001
60-69	2.59 (2.27,2.95)	<0.0001
70-79	3.56 (2.74,4.63)	<0.0001
80+	10.47 (6.35,17.26)	<0.0001
**Race/ethnicity**		
Hispanic, any race	0.81 (0.74,0.88)	<0.0001
Other/unknown	1.28 (1.13,1.44)	<0.0001
White, non-Hispanic	1.19 (1.02,1.39)	0.0231
Black, non-Hispanic	Reference	
**CD4+ T-cell count, cells/μL[1]**		
<200	1.52 (1.37,1.68)	<0.0001
200-349	1.13 (1.01,1.25)	0.03
350-499	1.08 (0.97,1.21)	0.17
≥500	Reference	
**HIV acquisition category**		
Injection drug use-related	1.86 (1.68,2.05)	<0.0001
Men who have sex with men	0.79 (0.69,0.91)	0.001
Other/unknown	1.34 (1.2,1.49)	<0.0001
Heterosexual	Reference	
**Health insurance type[1]**		
Public	1.97 (1.67,2.31)	<0.0001
Other/unknown	1.35 (1.13,1.62)	0.001
Commercial	Reference	

[1]At time of first achieving sustained virologic suppression.

Supplementary Table 3. Estimated mortality rate ratios in people with HIV in the Bronx, NY after first achieving sustained virologic suppression, stratified by sex.

	Female		Male		P-interaction
	**Relative Risk (95% CI)**	**P-value**	**Relative Risk (95% CI)**	**P-value**	
**Age, years[1]**					0.18
18-19	0.25 (0.09,0.67)	0.006	0.25 (0.10,0.61)	0.002	
20-29	0.42 (0.29,0.60)	<0.0001	0.26 (0.19,0.36)	<0.0001	
30-39	0.65 (0.54,0.79)	<0.0001	0.65 (0.55,0.76)	<0.0001	
40-49	Reference		Reference		
50-59	1.79 (1.54,2.08)	<0.0001	1.66 (1.47,1.86)	<0.0001	
60-69	2.40 (1.91,3.00)	<0.0001	2.58 (2.21,3.02)	<0.0001	
70-79	2.77 (1.78,4.32)	<0.0001	4.15 (3.02,5.72)	<0.0001	
80+	6.08 (2.46,15.03)	<0.0001	13.50 (7.39,24.67)	<0.0001	
**Race/ethnicity**					0.47
Hispanic, any race	0.84 (0.73,0.96)	0.01	0.80 (0.72,0.89)	<0.0001	
Other/unknown	1.19 (0.97,1.48)	0.10	1.32 (1.13,1.53)	0.0003	
White, non-Hispanic	1.37 (1.06,1.75)	0.02	1.13 (0.93,1.37)	0.22	
Black, non-Hispanic	Reference		Reference		
**CD4+ T-cell count, cells/µL[1]**					0.56
<200	1.61 (1.37,1.90)	<0.0001	1.49 (1.31,1.69)	<0.0001	
200-349	1.17 (0.99,1.39)	0.07	1.11 (0.97,1.27)	0.12	
350-499	1.03 (0.86,1.24)	0.73	1.12 (0.97,1.29)	0.12	
≥500	Reference		Reference		
**HIV acquisition category**					0.003
Injection drug use-related	2.30 (1.97,2.70)	<0.0001	1.66 (1.47,1.88)	<0.0001	
Men who have sex with men	NA		0.75 (0.64,0.87)	0.0001	
Other/unknown	1.47 (1.25,1.73)	<0.0001	1.23 (1.07,1.41)	0.005	
Heterosexual	Reference		Reference		
**Health insurance type[1]**					0.81
Public	1.81 (1.37,2.39)	<0.0001	2.06 (1.68,2.52)	<0.0001	
Other/unknown	1.27 (0.94,1.72)	0.12	1.48 (1.19,1.85)	0.0005	
Commercial	Reference		Reference		

[1]At time of first achieving sustained viral load suppression.

Supplementary Table 4. Mortality rate ratios compare males with females within each age group and were estimated from a multivariable Poisson model including age group, sex, race/ethnicity, HIV acquisition category, health insurance type, CD4+ T-cell count, and a sex-by-age interaction term, with log person-years as the offset. The overall sex-by-age interaction was not statistically significant (Wald p=0.18).

Age, years	Relative Risk (95% CI)	P-value
18-19	1.40 (0.38, 5.23)	0.61
20-29	0.89 (0.55, 1.43)	0.63
30-39	1.36 (1.10, 1.68)	0.004
40-49	1.32 (1.15, 1.51)	<0.0001
50-59	1.21 (1.06, 1.39)	0.006
60-69	1.45 (1.14, 1.83)	0.002
70-79	2.11 (1.25, 3.58)	0.005
80+	2.84 (0.98, 8.23)	0.06

Supplementary Figure 1. Flow diagram of inclusion and exclusion into the cohort.

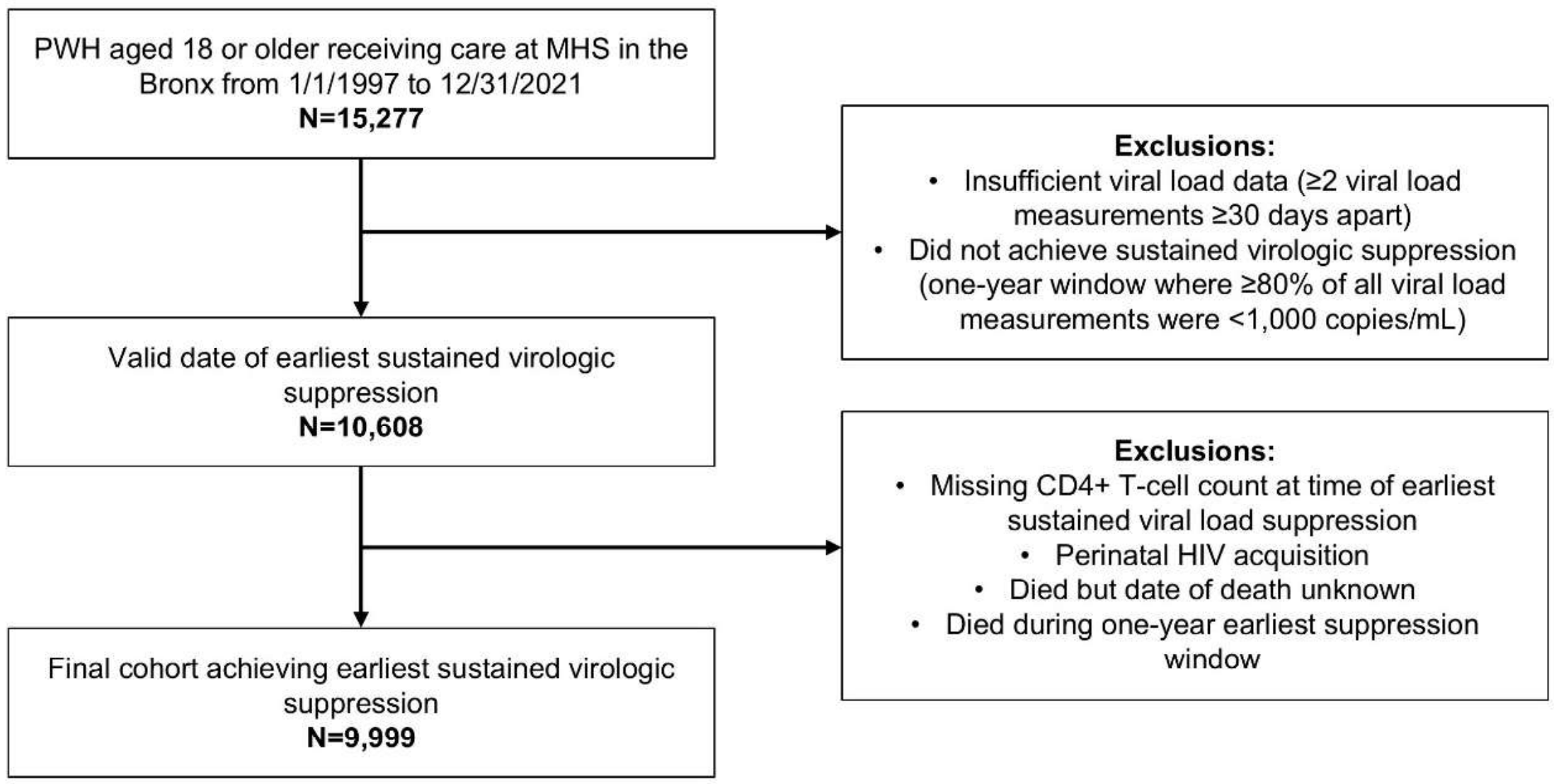

Supplementary Table 5. Estimated remaining years of life with 95% confidence interval for 30-year-old people with HIV after earliest sustained virologic suppression in the Bronx, NY, by sex, race/ethnicity, acquisition risk, and CD4+ T-cell count and health insurance at time of earliest sustained virologic suppression.

Sex	Race_Ethnicity	CD4_count	Acquisition risk	Insurance	sample.size	deaths	RLY	RLYs_lower	RLYs_upper
Female	Black	>=500	Heterosexual	Public Insurance	576	69	41.59	40.14	43.01
Female	Hispanic	>=500	Heterosexual	Public Insurance	501	43	44.59	43.15	46.1
Male	Hispanic	>=500	MSM	Public Insurance	411	23	44.15	42.16	45.87
Male	Black	>=500	MSM	Public Insurance	379	30	41.15	39.25	43.09
Male	Hispanic	>=500	Heterosexual	Public Insurance	218	38	40.78	39.15	42.48
Male	Black	>=500	Heterosexual	Public Insurance	203	41	37.61	35.86	39.29
Female	Black	350-499	Heterosexual	Public Insurance	156	30	40.32	38.6	42.04
Female	Black	>=500	Heterosexual	Unknown	148	26	46.28	44.55	47.91
Female	Hispanic	350-499	Heterosexual	Public Insurance	140	22	43.37	41.66	45.12
Female	Black	200-349	Heterosexual	Public Insurance	137	37	39.7	38.09	41.35
Male	Black	350-499	MSM	Public Insurance	135	10	39.96	38	42.13
Male	Hispanic	>=500	IDU	Public Insurance	128	34	31.51	29.82	33.26
Male	Hispanic	350-499	MSM	Public Insurance	120	11	43.04	40.92	45.11
Female	Black	<200	Heterosexual	Public Insurance	116	59	35.23	33.75	36.73
Male	Black	<200	Heterosexual	Public Insurance	115	64	31.31	29.73	32.89
Male	Black	350-499	Heterosexual	Public Insurance	114	35	36.38	34.51	38.1
Male	Hispanic	<200	IDU	Public Insurance	113	80	25.68	24.09	27.4
Female	Hispanic	>=500	Heterosexual	Unknown	110	4	48.93	47.37	50.45
Female	Black	>=500	Other/Unknown	Public Insurance	110	21	37.32	35.49	39.12
Female	Hispanic	200-349	Heterosexual	Public Insurance	106	37	42.8	41.21	44.39
Female	Hispanic	<200	Heterosexual	Public Insurance	106	51	38.43	36.73	40.1
Male	Black	200-349	Heterosexual	Public Insurance	105	40	35.72	33.95	37.46
Male	Black	200-349	MSM	Public Insurance	98	26	39.28	37.1	41.27
Male	Black	>=500	Other/Unknown	Public Insurance	98	17	33.41	31.53	35.29
Male	Hispanic	350-499	Heterosexual	Public Insurance	97	23	39.56	37.76	41.16
Male	Hispanic	200-349	IDU	Public Insurance	97	49	29.75	28.05	31.47
Male	Other	>=500	MSM	Public Insurance	96	7	37.61	35.25	40.16
Male	Hispanic	<200	Heterosexual	Public Insurance	96	51	34.42	32.79	36.08
Female	Black	>=500	Other/Unknown	Unknown	94	12	42.29	40.45	44.19
Male	Hispanic	200-349	Heterosexual	Public Insurance	91	26	38.89	37.11	40.7
Male	Hispanic	200-349	MSM	Public Insurance	90	21	42.38	40.41	44.46
Female	Black	>=500	Heterosexual	Commercial	87	6	50.33	48.02	52.52
Female	Other	>=500	Heterosexual	Public Insurance	86	10	38.1	36.17	40.24
Male	Hispanic	>=500	Other/Unknown	Public Insurance	86	18	36.52	34.57	38.51
Male	Black	<200	MSM	Public Insurance	84	25	34.89	32.93	36.99
Male	Black	>=500	MSM	Commercial	80	0	50.05	47.69	52.33
Female	Hispanic	>=500	Other/Unknown	Public Insurance	78	8	40.51	38.54	42.48
Male	Hispanic	<200	MSM	Public Insurance	75	29	38.01	36	40.03
Male	Hispanic	350-499	IDU	Public Insurance	73	26	30.32	28.63	32.08
Male	Black	>=500	IDU	Public Insurance	71	25	28.54	26.99	30.19
Male	Black	<200	IDU	Public Insurance	70	54	23.08	21.26	24.69
Male	Black	>=500	Other/Unknown	Unknown	69	11	38.39	36.51	40.15
Female	Hispanic	>=500	IDU	Public Insurance	69	18	35.49	33.56	37.45
Male	Hispanic	>=500	MSM	Commercial	64	0	52.31	49.95	54.49
Male	Hispanic	>=500	MSM	Unknown	63	3	48.54	46.4	50.63
Male	Other	>=500	Heterosexual	Public Insurance	62	15	34.06	31.97	36.18
Male	Hispanic	350-499	Other/Unknown	Public Insurance	55	15	35.3	33.28	37.47
Female	Black	>=500	IDU	Public Insurance	55	27	32.29	30.51	34.17
Male	Black	200-349	IDU	Public Insurance	52	31	26.84	25.29	28.49
Female	Hispanic	>=500	Heterosexual	Commercial	51	5	52.59	50.71	54.43
Male	Hispanic	>=500	Heterosexual	Unknown	51	8	45.47	43.64	47.35
Male	Black	>=500	MSM	Unknown	50	3	45.97	43.75	48.15
Male	Hispanic	<200	Other/Unknown	Public Insurance	50	33	30.3	28.32	32.24
Male	Black	>=500	Heterosexual	Commercial	49	9	47.09	44.67	49.58
Male	Black	<200	Other/Unknown	Public Insurance	49	31	27.32	25.55	29.11
Male	Black	>=500	Heterosexual	Unknown	45	14	42.55	40.68	44.52
Male	Hispanic	>=500	Other/Unknown	Unknown	44	8	41.51	39.57	43.42
Male	Other	>=500	Other/Unknown	Public Insurance	44	13	29.89	27.81	31.98

Male	Black	350-499	IDU	Public Insurance	44	22	27.44	25.8	29.34
Female	Hispanic	>=500	Other/Unknown	Unknown	42	9	45.29	43.23	47.2
Male	White	>=500	MSM	Public Insurance	42	8	38.39	35.56	41.15
Male	Black	200-349	Other/Unknown	Public Insurance	42	22	31.53	29.66	33.49
Male	Black	>=500	Other/Unknown	Commercial	40	4	43.15	40.59	45.77
Male	Other	>=500	Other/Unknown	Unknown	40	6	34.81	32.66	36.97
Female	Hispanic	<200	IDU	Public Insurance	40	27	29.28	27.29	31.26
Female	Black	>=500	Other/Unknown	Commercial	38	2	46.85	44.29	49.18
Female	Black	350-499	Heterosexual	Unknown	38	8	45.21	43.33	47.01
Male	Black	350-499	Other/Unknown	Unknown	38	12	37.17	35.06	39.53
Female	Black	200-349	Other/Unknown	Public Insurance	38	15	35.52	33.49	37.44
Female	Hispanic	350-499	IDU	Public Insurance	35	14	34.24	32.07	36.26
Female	Other	>=500	Other/Unknown	Unknown	34	2	38.78	36.51	41.07
Female	Hispanic	350-499	Other/Unknown	Public Insurance	33	11	39.29	37.06	41.42
Male	Hispanic	>=500	IDU	Unknown	33	12	36.5	34.57	38.55
Male	Black	350-499	Other/Unknown	Public Insurance	32	16	32.17	30.19	34.13
Female	Black	<200	Other/Unknown	Public Insurance	32	18	31.11	29.17	33.05
Female	Hispanic	200-349	Heterosexual	Unknown	31	9	47.32	45.54	49.04
Female	White	>=500	Heterosexual	Public Insurance	31	10	38.78	36.28	41.38
Female	Black	350-499	Other/Unknown	Public Insurance	31	6	36.14	33.98	38.44
Male	Hispanic	200-349	Other/Unknown	Public Insurance	31	13	34.57	32.66	36.64
Female	Hispanic	<200	Other/Unknown	Public Insurance	31	17	34.19	32.04	36.42
Female	Other	>=500	Other/Unknown	Public Insurance	31	4	33.78	31.65	36.1
Male	White	>=500	Other/Unknown	Unknown	30	7	35.55	32.94	38.51
Male	Other	200-349	Heterosexual	Public Insurance	30	17	32.18	29.97	34.51
Male	Hispanic	>=500	Heterosexual	Commercial	29	0	49.7	47.34	51.87
Female	Other	200-349	Heterosexual	Public Insurance	29	10	36.18	33.94	38.41
Female	Other	>=500	Heterosexual	Unknown	28	4	42.93	40.74	45.11
Male	Black	200-349	Other/Unknown	Unknown	28	14	36.42	34.42	38.48
Female	Hispanic	200-349	IDU	Public Insurance	28	18	33.57	31.75	35.48
Male	Hispanic	<200	IDU	Unknown	28	18	30.23	28.21	32.29
Male	Other	>=500	IDU	Public Insurance	28	13	25.4	23.25	27.47
Male	Black	350-499	Heterosexual	Unknown	27	8	41.36	39.22	43.42
Female	Other	350-499	Heterosexual	Public Insurance	27	3	36.77	34.39	38.96
Male	Other	350-499	Heterosexual	Public Insurance	27	10	32.89	30.61	35.01
Male	Other	>=500	MSM	Commercial	26	2	47.05	44.07	49.77
Male	Hispanic	350-499	IDU	Unknown	26	15	35.3	33.16	37.41
Male	White	>=500	Heterosexual	Public Insurance	26	10	34.8	32.21	37.35
Male	Hispanic	200-349	IDU	Unknown	26	15	34.57	32.56	36.68
Female	Black	<200	IDU	Public Insurance	26	22	26.43	24.7	28.37
Male	Other	<200	IDU	Public Insurance	26	24	20.28	18.4	22.17
Male	Black	>=500	IDU	Unknown	25	11	33.35	31.26	35.37
Male	Other	<200	Other/Unknown	Public Insurance	25	13	24.3	22.33	26.29
Female	Hispanic	350-499	Heterosexual	Unknown	24	6	47.93	46.07	49.77
Male	Black	350-499	MSM	Unknown	24	5	44.77	42.34	47.06
Female	Black	200-349	Heterosexual	Unknown	24	9	44.55	42.73	46.44
Female	Hispanic	>=500	IDU	Unknown	24	6	40.47	38.35	42.58
Male	Other	350-499	MSM	Public Insurance	24	4	36.36	33.6	39.19
Male	Hispanic	>=500	Other/Unknown	Commercial	23	2	46.13	43.57	48.56
Male	Black	200-349	MSM	Commercial	21	6	48.5	46.04	51.16
Male	Hispanic	200-349	Other/Unknown	Unknown	21	8	39.64	37.43	41.92
Female	Black	200-349	IDU	Public Insurance	21	12	30.47	28.54	32.39
Male	Hispanic	350-499	MSM	Commercial	20	2	51.41	49.07	53.68
Male	Black	350-499	Heterosexual	Commercial	20	5	45.98	43.52	48.57
Female	Hispanic	<200	Heterosexual	Unknown	20	12	43.32	41.38	45.43
Male	Black	200-349	Heterosexual	Unknown	20	7	40.71	38.68	42.72
Female	Black	<200	Heterosexual	Unknown	20	8	40.26	38.23	42.25
Male	Black	200-349	Heterosexual	Commercial	19	5	45.42	42.91	47.93
Male	Black	<200	Heterosexual	Unknown	19	12	36.25	34.26	38.29
Male	Black	<200	Other/Unknown	Unknown	19	10	32.07	30.08	34.1
Female	Other	<200	Heterosexual	Public Insurance	19	11	31.78	29.59	33.88
Male	Other	<200	MSM	Public Insurance	19	12	31.3	29.05	33.75

Male	Other	<200	Heterosexual	Public Insurance	19	17	27.96	26.05	29.95
Female	Black	350-499	Heterosexual	Commercial	18	1	49.32	47.04	51.64
Male	Hispanic	350-499	MSM	Unknown	18	1	47.58	45.42	49.65
Female	Black	200-349	Other/Unknown	Unknown	18	7	40.45	38.49	42.52
Male	Black	350-499	IDU	Unknown	18	11	32.07	30	34.19
Female	Black	350-499	IDU	Public Insurance	18	8	31.13	29.15	33.08
Male	Other	200-349	Other/Unknown	Public Insurance	18	8	28.18	25.97	30.69
Male	White	>=500	MSM	Commercial	17	3	47.7	44.7	50.5
Female	Black	<200	Heterosexual	Commercial	17	9	45.04	42.59	47.41
Female	Black	350-499	Other/Unknown	Unknown	17	5	41.13	39.07	43.21
Female	Black	200-349	Heterosexual	Commercial	16	6	48.85	46.52	51.08
Male	Hispanic	350-499	Heterosexual	Unknown	16	2	44.39	42.3	46.37
Male	Black	<200	Heterosexual	Commercial	16	4	41.2	38.52	43.94
Male	Black	<200	MSM	Unknown	16	9	39.83	37.5	42.16
Male	Other	>=500	Other/Unknown	Commercial	16	2	39.75	36.88	42.79
Male	Hispanic	<200	Heterosexual	Unknown	16	9	39.44	37.4	41.69
Female	Hispanic	200-349	Other/Unknown	Public Insurance	16	10	38.62	36.56	40.77
Female	Black	>=500	IDU	Unknown	16	3	37.25	35.16	39.35
Female	White	<200	Heterosexual	Public Insurance	16	9	32.55	30.02	35.16
Male	Other	350-499	IDU	Public Insurance	16	7	24.29	22.29	26.36
Male	Black	350-499	MSM	Commercial	15	3	49.08	46.52	51.65
Female	Other	>=500	Heterosexual	Commercial	15	2	47.46	44.9	50.09
Male	Hispanic	200-349	Heterosexual	Unknown	15	5	43.71	41.82	45.72
Male	Hispanic	350-499	Other/Unknown	Unknown	15	2	40.3	38.22	42.5
Male	Hispanic	<200	Other/Unknown	Unknown	15	7	35.17	33.14	37.19
Male	White	>=500	Other/Unknown	Public Insurance	15	4	30.65	28.34	33.17
Male	Other	200-349	IDU	Public Insurance	15	12	23.79	21.86	25.86
Female	Hispanic	350-499	Other/Unknown	Unknown	14	2	44.11	42.09	46.14
Male	Other	>=500	MSM	Unknown	14	1	42.57	39.92	45.16
Female	Black	<200	Other/Unknown	Unknown	14	12	36.02	33.95	38.25
Male	Other	350-499	Other/Unknown	Public Insurance	14	4	28.81	26.45	31.2
Male	White	<200	IDU	Public Insurance	14	12	20.91	18.86	23.08
Male	Black	200-349	MSM	Unknown	13	4	44.13	41.71	46.43
Male	White	350-499	MSM	Public Insurance	13	3	37.14	34.58	40.11
Male	Black	200-349	IDU	Unknown	13	8	31.5	29.51	33.53
Male	Other	<200	Other/Unknown	Unknown	13	7	28.71	26.69	30.95
Male	Hispanic	350-499	Heterosexual	Commercial	12	2	48.66	46.17	51.23
Male	Other	>=500	Heterosexual	Commercial	12	0	43.72	40.79	46.65
Male	Black	200-349	Other/Unknown	Commercial	12	2	41.34	38.52	44.06
Female	White	350-499	Heterosexual	Public Insurance	12	3	37.57	35.02	40.39
Female	White	200-349	Heterosexual	Public Insurance	12	6	36.95	34.53	39.39
Male	White	200-349	Heterosexual	Public Insurance	12	5	32.95	30.54	35.59
Male	White	>=500	IDU	Public Insurance	12	5	26.06	23.68	28.39
Female	Hispanic	200-349	Heterosexual	Commercial	11	4	51.33	49.11	53.26
Male	Hispanic	200-349	MSM	Unknown	11	3	46.99	44.71	49.09
Male	White	>=500	MSM	Unknown	11	1	43.37	40.58	46.21
Male	Other	>=500	Heterosexual	Unknown	11	3	39.07	36.76	41.49
Female	Hispanic	200-349	IDU	Unknown	11	3	38.66	36.34	40.82
Male	Other	200-349	MSM	Public Insurance	11	5	35.79	33.31	38.48
Male	White	350-499	Heterosexual	Public Insurance	11	4	33.58	30.91	36.26
Female	Other	>=500	IDU	Public Insurance	11	6	28.9	26.7	31.11
Female	White	<200	IDU	Public Insurance	11	9	24.06	21.68	26.39
Female	Other	<200	IDU	Public Insurance	11	8	23.47	21.41	25.56
Male	Hispanic	200-349	Heterosexual	Commercial	10	3	48.14	45.61	50.47
Male	Black	<200	MSM	Commercial	10	4	44.63	41.74	47.32
Female	Other	>=500	Other/Unknown	Commercial	10	1	43.58	40.74	46.58
Male	Hispanic	<200	MSM	Unknown	10	3	42.93	40.52	45.26
Male	Black	350-499	Other/Unknown	Commercial	10	2	42.05	39.57	44.57
Male	White	200-349	MSM	Public Insurance	10	2	36.49	33.68	39.35
Female	Hispanic	<200	IDU	Unknown	10	4	34.13	31.91	36.54
Female	Hispanic	>=500	Other/Unknown	Commercial	9	1	49.46	46.87	51.93
Male	Hispanic	<200	Heterosexual	Commercial	9	3	44.22	41.65	46.64

Male	Hispanic	>=500	IDU	Commercial	9	3	41.34	38.5	44.32
Female	Hispanic	350-499	IDU	Unknown	9	3	39.21	36.96	41.68
Female	White	>=500	Other/Unknown	Public Insurance	9	2	34.64	32.09	37.31
Male	White	200-349	Other/Unknown	Public Insurance	9	5	28.79	26.32	31.48
Female	Other	350-499	IDU	Public Insurance	9	7	27.78	25.59	30.06
Female	Other	<200	Other/Unknown	Public Insurance	9	6	27.74	25.4	30.01
Male	Black	<200	IDU	Unknown	9	7	27.35	25.4	29.27
Male	White	350-499	IDU	Public Insurance	9	3	24.98	22.85	27.42
Male	White	<200	Other/Unknown	Public Insurance	9	5	24.97	22.73	27.35
Male	Hispanic	350-499	Other/Unknown	Commercial	8	2	44.94	42.06	47.74
Female	White	>=500	Other/Unknown	Unknown	8	2	39.53	36.82	42.31
Female	Hispanic	<200	Other/Unknown	Unknown	8	5	39.2	36.99	41.66
Male	Other	200-349	Heterosexual	Unknown	8	4	37.16	34.58	39.67
Male	Black	<200	Other/Unknown	Commercial	8	5	36.92	34.11	39.82
Female	Other	<200	Heterosexual	Unknown	8	3	36.71	34.36	39.13
Male	Other	350-499	Other/Unknown	Unknown	8	3	33.61	31.39	35.84
Female	Other	350-499	Other/Unknown	Public Insurance	8	2	32.64	30.12	35.02
Male	White	350-499	Other/Unknown	Public Insurance	8	4	29.41	26.96	31.97
Female	White	<200	Other/Unknown	Public Insurance	8	6	28.43	25.85	31.06
Male	White	200-349	IDU	Public Insurance	8	8	24.38	22.13	26.69
Female	Hispanic	<200	Heterosexual	Commercial	7	3	47.78	45.35	50.22
Female	Black	350-499	Other/Unknown	Commercial	7	1	45.74	43.11	48.3
Female	White	>=500	Heterosexual	Unknown	7	1	43.67	41.27	46.22
Female	Hispanic	200-349	Other/Unknown	Unknown	7	1	43.46	41.24	45.62
Female	Other	350-499	Heterosexual	Unknown	7	1	41.78	39.29	44.1
Male	Hispanic	350-499	IDU	Commercial	7	2	40.21	37.19	43.19
Female	Other	200-349	Other/Unknown	Unknown	7	4	36.93	34.63	39.27
Male	White	<200	MSM	Public Insurance	7	1	32.16	29.33	34.86
Male	Other	>=500	IDU	Unknown	7	4	29.89	27.67	32.04
Female	White	>=500	IDU	Public Insurance	7	2	29.64	27.29	32.29
Male	White	<200	Heterosexual	Public Insurance	7	4	28.66	26.25	31.19
Female	Other	200-349	IDU	Public Insurance	7	4	27.21	24.92	29.45
Male	Hispanic	200-349	Other/Unknown	Commercial	6	3	44.48	41.8	47.18
Male	Other	350-499	Heterosexual	Commercial	6	0	42.73	39.67	45.7
Female	Other	200-349	Heterosexual	Unknown	6	3	41.13	38.68	43.52
Male	Other	<200	Heterosexual	Commercial	6	1	37.55	34.82	40.43
Female	Other	<200	Other/Unknown	Unknown	6	3	32.45	30.04	35.01
Female	Other	200-349	Other/Unknown	Public Insurance	6	3	31.92	29.63	34.25
Male	Hispanic	200-349	MSM	Commercial	5	1	51	48.62	53.32
Female	White	>=500	Heterosexual	Commercial	5	0	48.11	45.15	50.93
Male	Hispanic	<200	MSM	Commercial	5	2	47.39	44.8	49.97
Male	White	>=500	Heterosexual	Commercial	5	0	44.57	41.5	47.87
Female	White	200-349	Heterosexual	Unknown	5	3	41.95	39.29	44.52
Male	Other	350-499	MSM	Unknown	5	1	41.4	38.62	44.1
Female	Black	<200	Other/Unknown	Commercial	5	3	40.91	38.07	43.66
Male	Other	200-349	MSM	Unknown	5	2	40.8	38.04	43.62
Male	White	>=500	Heterosexual	Unknown	5	0	39.84	36.95	42.49
Male	Black	>=500	IDU	Commercial	5	2	38.32	35.51	41.25
Female	Other	350-499	Other/Unknown	Unknown	5	2	37.54	34.94	39.96
Male	Black	350-499	IDU	Commercial	5	1	37.02	33.97	40.07
Male	Hispanic	<200	IDU	Commercial	5	4	35	32.1	38.09
Male	White	350-499	Other/Unknown	Unknown	5	3	34.44	31.86	37.23
Female	White	350-499	Other/Unknown	Public Insurance	5	1	33.38	30.58	36.2
Male	Other	200-349	Other/Unknown	Unknown	5	0	32.93	30.61	35.23
Female	White	200-349	Other/Unknown	Public Insurance	5	3	32.72	29.92	35.5
Female	Black	<200	IDU	Unknown	5	5	31.03	28.88	33.25
Male	White	>=500	IDU	Unknown	5	3	30.61	28.09	33.22
Female	White	350-499	IDU	Public Insurance	5	2	28.54	26.05	31.27
Female	Hispanic	200-349	Other/Unknown	Commercial	4	1	47.89	45.39	50.25
Male	White	350-499	MSM	Commercial	4	1	46.63	43.26	49.58
Male	Other	350-499	MSM	Commercial	4	0	45.97	42.88	48.85
Female	Black	200-349	Other/Unknown	Commercial	4	0	45.21	42.78	47.84

Female	White	>=500	Other/Unknown	Commercial	4	0	44.27	41.17	47.43
Female	Other	350-499	Other/Unknown	Commercial	4	0	42.44	39.59	45.6
Male	White	350-499	MSM	Unknown	4	1	42.13	39.17	44.97
Male	Other	200-349	Heterosexual	Commercial	4	1	41.96	38.92	45.06
Male	White	200-349	MSM	Unknown	4	3	41.47	38.65	44.37
Female	Black	350-499	IDU	Unknown	4	1	36.11	33.63	38.42
Female	White	200-349	IDU	Unknown	4	3	32.6	30.02	35.29
Male	Black	<200	IDU	Commercial	4	3	31.99	29.24	34.62
Male	Other	350-499	IDU	Unknown	4	1	28.77	26.38	31.33
Male	Other	<200	IDU	Unknown	4	4	24.28	22.15	26.42
Female	Hispanic	350-499	Heterosexual	Commercial	3	0	51.75	49.56	53.8
Female	Hispanic	350-499	Other/Unknown	Commercial	3	1	48.51	46.03	50.89
Female	White	350-499	Heterosexual	Commercial	3	0	47.07	43.88	49.97
Male	Other	200-349	MSM	Commercial	3	0	45.41	42.33	48.29
Male	White	200-349	Heterosexual	Commercial	3	1	42.7	39.46	45.98
Male	Other	<200	MSM	Commercial	3	1	41.14	38.03	44.43
Male	White	>=500	Other/Unknown	Commercial	3	1	40.45	37.19	43.58
Male	White	200-349	Other/Unknown	Commercial	3	0	38.61	35.43	41.86
Male	Other	350-499	Other/Unknown	Commercial	3	0	38.47	35.64	41.52
Male	Other	200-349	Other/Unknown	Commercial	3	0	37.85	34.83	40.87
Male	Other	350-499	Heterosexual	Unknown	3	0	37.79	35.33	40.15
Female	White	200-349	Other/Unknown	Unknown	3	1	37.72	35.06	40.26
Female	Black	200-349	IDU	Unknown	3	3	35.47	33.25	37.65
Male	Other	<200	Heterosexual	Unknown	3	2	32.7	30.34	35.04
Male	White	200-349	IDU	Unknown	3	3	28.81	26.27	31.49
Male	Other	200-349	IDU	Unknown	3	3	28.12	25.77	30.4
Female	White	200-349	IDU	Public Insurance	3	2	27.86	25.38	30.43
Male	White	<200	IDU	Unknown	3	3	24.85	22.63	27.13
Female	Other	350-499	Heterosexual	Commercial	2	1	46.36	43.6	48.95
Female	Hispanic	<200	Other/Unknown	Commercial	2	0	43.95	41.04	46.84
Female	White	<200	Heterosexual	Commercial	2	2	42.25	38.88	45.56
Female	Black	>=500	IDU	Commercial	2	0	42.24	39.43	44.91
Male	Hispanic	<200	Other/Unknown	Commercial	2	1	40.13	37.33	42.99
Male	Hispanic	200-349	IDU	Commercial	2	1	39.5	36.44	42.44
Male	White	350-499	Heterosexual	Unknown	2	1	38.5	35.58	41.3
Female	White	350-499	Other/Unknown	Unknown	2	1	38.41	35.66	41.27
Female	White	<200	Heterosexual	Unknown	2	1	37.51	34.7	40.31
Female	Other	<200	Other/Unknown	Commercial	2	0	37.36	34.19	40.61
Male	Black	200-349	IDU	Commercial	2	1	36.32	33.6	39.4
Male	Other	<200	MSM	Unknown	2	2	36.24	33.37	39.07
Female	Black	<200	IDU	Commercial	2	1	36.01	32.95	39
Female	White	>=500	IDU	Unknown	2	0	34.51	31.54	37.42
Male	White	<200	Other/Unknown	Commercial	2	2	34.2	31.02	37.55
Female	Other	>=500	IDU	Unknown	2	2	33.81	31.36	36.35
Male	White	200-349	Other/Unknown	Unknown	2	2	33.68	31.14	36.46
Male	White	<200	Heterosexual	Unknown	2	2	33.49	30.78	36.46
Male	Other	350-499	IDU	Commercial	2	1	33.41	30.36	36.64
Female	White	<200	Other/Unknown	Unknown	2	2	33.28	30.41	35.95
Female	Other	<200	IDU	Unknown	2	1	27.72	25.41	30.22
Female	Other	200-349	Heterosexual	Commercial	1	1	45.72	42.91	48.67
Male	White	350-499	Heterosexual	Commercial	1	0	43.44	40.22	46.7
Female	Hispanic	200-349	IDU	Commercial	1	1	43.43	40.22	46.49
Female	White	350-499	Other/Unknown	Commercial	1	0	43.12	39.76	46.58
Female	White	350-499	Heterosexual	Unknown	1	1	42.51	39.97	45.24
Female	Other	200-349	Other/Unknown	Commercial	1	1	41.81	38.74	44.99
Female	Other	<200	Heterosexual	Commercial	1	0	41.61	38.76	44.74
Female	White	>=500	IDU	Commercial	1	0	39.55	36.06	43.18
Male	White	350-499	Other/Unknown	Commercial	1	0	39.29	35.83	42.45
Female	Other	>=500	IDU	Commercial	1	0	38.7	35.56	41.95
Male	White	<200	Heterosexual	Commercial	1	0	38.42	35.07	41.91
Female	White	350-499	IDU	Commercial	1	0	38.12	34.59	41.77
Male	White	200-349	Heterosexual	Unknown	1	1	38.02	35.19	41

Female	Other	200-349	IDU	Commercial	1	0	36.76	33.49	40.03
Male	Other	>=500	IDU	Commercial	1	0	34.72	31.75	37.67
Male	Other	<200	Other/Unknown	Commercial	1	1	33.36	30.6	36.36
Female	White	350-499	IDU	Unknown	1	1	33.33	30.48	36.44
Female	Other	350-499	IDU	Unknown	1	1	32.57	30.05	35.29
Female	Other	<200	IDU	Commercial	1	1	32.36	29.11	35.89
Female	Other	200-349	IDU	Unknown	1	0	31.92	29.46	34.67
Female	White	<200	IDU	Unknown	1	1	28.49	25.88	31.35
Male	Other	<200	IDU	Commercial	1	1	28.49	25.48	31.61
Female	White	200-349	Heterosexual	Commercial	0	0	46.44	43.42	49.31
Male	White	200-349	MSM	Commercial	0	0	46.16	42.92	49.07
Female	Hispanic	>=500	IDU	Commercial	0	0	45.17	42.41	48.06
Female	Hispanic	350-499	IDU	Commercial	0	0	44.05	41.27	46.94
Female	White	200-349	Other/Unknown	Commercial	0	0	42.67	39.24	46.03
Male	White	<200	MSM	Commercial	0	0	41.88	38.47	45.37
Female	Black	350-499	IDU	Commercial	0	0	41	38.17	43.86
Female	Black	200-349	IDU	Commercial	0	0	40.33	37.3	43.43
Female	Hispanic	<200	IDU	Commercial	0	0	39.08	36.27	42.02
Female	White	<200	Other/Unknown	Commercial	0	0	38.11	34.69	41.51
Female	White	200-349	IDU	Commercial	0	0	37.62	33.9	41.14
Female	Other	350-499	IDU	Commercial	0	0	37.54	34.28	40.96
Male	White	<200	MSM	Unknown	0	0	37.05	34.14	39.88
Male	White	>=500	IDU	Commercial	0	0	35.41	31.87	39.01
Male	White	350-499	IDU	Commercial	0	0	34.24	30.95	37.7
Male	White	200-349	IDU	Commercial	0	0	33.61	30.23	37.18
Female	White	<200	IDU	Commercial	0	0	33.17	29.68	36.88
Male	Other	200-349	IDU	Commercial	0	0	32.83	29.83	36.11
Male	White	350-499	IDU	Unknown	0	0	29.47	26.98	32.2
Male	White	<200	Other/Unknown	Unknown	0	0	29.44	26.89	31.98
Male	White	<200	IDU	Commercial	0	0	29.3	26.16	32.71
